# Factors Associated With the Quality of Staff-Resident Interactions in Assisted Living

**DOI:** 10.1093/geroni/igab046.268

**Published:** 2021-12-17

**Authors:** Anju Paudel, Elizabeth Galik, Barbara Resnick, Kelly Doran, Marie Boltz, Shijun Zhu

**Affiliations:** 1 Penn State Ross and Carol Nese College of Nursing, University Park, Pennsylvania, United States; 2 University of Maryland School of Nursing, Baltimore, Maryland, United States; 3 Pennsylvania State University, University Park, Pennsylvania, United States; 4 University of Maryland, Baltimore, Maryland, United States

## Abstract

Care interactions are essential to understand and respond to resident needs in assisted living (AL). The factors that influence care interactions in AL have not been directly examined. In this study, we explored the factors associated with the quality of care interactions in AL. It was hypothesized that resident functional status, agitation, depression, and resistiveness to care as well as facility size and ownership would be significantly associated with the quality of care interactions in AL after controlling for resident demographics (age, gender, marital status), comorbidities, and cognition. To test the hypothesis, we utilized baseline data including 379 residents from the second and third cohorts recruited in a randomized trial titled ‘Dissemination and Implementation of Function Focused Care for Assisted Living Using the Evidence Integration Triangle’. Regression analysis was performed using a stepwise method. The care interactions were mostly positive (mean=6.3; range = 0-7). Resident agitation and facility ownership were significantly associated with care interactions and accounted for 8.2% of the variance. Increased resident agitation was associated with negative or neutral interaction while for-profit ownership was associated with positive interactions. To promote positive care interactions, staff should be educated about strategies to minimize resident agitation (e.g., calm posture and respectful listening) and encouraged to engage with residents using resident-centered care and communication approach. Findings also suggest the need to work towards optimizing care interactions in nonprofit stings. Future research could further explore the impact of facility-level factors (e.g., staffing ratios, staff longevity, and job satisfaction) on care interactions.

